# Public Health Risks from Mismanagement of Healthcare Wastes in Shinyanga Municipality Health Facilities, Tanzania

**DOI:** 10.1155/2015/981756

**Published:** 2015-12-08

**Authors:** Kizito Kuchibanda, Aloyce W. Mayo

**Affiliations:** Department of Water Resources Engineering, University of Dar es Salaam, P.O. Box 35136, Dar es Salaam, Tanzania

## Abstract

The increase of healthcare facilities in Shinyanga municipality has resulted in an increase of healthcare wastes, which poses serious threats to the environment, health workers, and the general public. This research was conducted to investigate management practices of healthcare wastes in Shinyanga municipality with a view of assessing health risks to health workers and the general public. The study, which was carried out in three hospitals, involved the use of questionnaires, in-depth interview, and observation checklist. The results revealed that healthcare wastes are not quantified or segregated in all the three hospitals. Healthcare wastes at the Shinyanga Regional Referral Hospital are disposed of by on-site incineration and burning and some wastes are disposed off-site. At Kolandoto DDH only on-site burning and land disposal are practiced, while at Kambarage UHC healthcare solid wastes are incinerated, disposed of on land disposal, and burned. Waste management workers do not have formal training in waste management techniques and the hospital administrations pay very little attention to appropriate management of healthcare wastes. In light of this, it is evident that management of healthcare solid wastes is not practiced in accordance with the national and WHO's recommended standards.

## 1. Introduction

Healthcare facilities generate healthcare wastes, which are of great importance due to their potential environmental hazards and public health risks [1]. Pollutants from healthcare units include biohazardous waste, chemicals, pharmaceutical wastes, pathological wastes, radioactive substances, and genotoxic wastes, which can cause a variety of adverse effects on human beings and the environment [1–3]. As a result, World Health Organization (WHO) has considered healthcare wastes as special wastes and it is now commonly acknowledged that certain categories of healthcare wastes are among the most hazardous and potentially dangerous of all wastes arising in communities [4].

In recent years the world has experienced a dramatic increase in the amount of hazardous waste generated, which was accompanied with vigorous drive for sustainable development and increased awareness and concern for the environment [5]. The developing world has had to grapple with managing this type of waste against the backdrop of competing priorities such as the HIV/AIDS pandemic. Incidentally, it is also the developing world that has been affected the most by the pandemic [6]. As a result of the high HIV/AIDS prevalence in this part of the world, there is a considerable rise in hospital admissions and a high morbidity among the general population.

About 10–25% of the volume of healthcare waste from hospitals and healthcare institutions worldwide presents a serious health hazard to patients, healthcare workers, and anybody who comes in contact with it [7, 8]. The hazardous wastes are those that may cause ill health or increase mortality in humans, fauna, and flora or adversely affect the environment when improperly treated, stored, transported, or disposed of [9–11]. Hazardous wastes are normally produced in labor wards, operation theatres, and laboratories [12]. The remaining 75% to 90%, which is generated from offices, kitchens, and housekeeping sections, is non-hazardous and poses no risk of infection transmission, as it is comparable to domestic wastes [4]. Thus, if the wastes are not segregated properly at the point of generation there will be a mixture of all these items plus kitchen wastes, office wastes, and floor wastes, which do not arise as a result of patients being attended [13].

Several scholars have suggested that planning and implementation of waste management can reduce health and environmental risks [10, 14]. In addition, good healthcare waste management in a hospital depends on a dedicated waste management team, good administration, careful planning, sound organization, underpinning legislation, adequate financing, and full participation by trained staff [7]. In the context of hospitals, segregation or separation of wastes is a very important stage in the waste management processes [15]. This enables those who handle the containers outside the hospital wards to identify and treat them appropriately [4]. Healthcare waste management is important to ensure proper hygiene in the health institutions and safety of healthcare workers and communities [16–18]. They require proper treatment to reduce direct exposure and to become less dangerous to humans, at recovering recyclable materials and at protecting the environment [9, 19]. Other scholars have recommended adoption of required guidelines and punitive compulsion and increased government responsibility for improving management of healthcare wastes [20].

In developing countries, healthcare solid wastes have not received sufficient attention because, very often, health issues compete with other sectors of the economy for the very limited resources available. Therefore, management of healthcare waste ends up not getting the priority it deserves [15, 21]. In Tanzania, hazardous and healthcare solid wastes are still handled and disposed of together with domestic wastes, thus creating a potential public health risk and an environmental burden [15, 22]. The Ministry of Health (MoH) and the World Health Organization (WHO) conducted a survey in the year 2000 to study the management of the syringes and needles used during immunization programmes in Tanzania [13]. This was followed by a similar survey on the management of all healthcare wastes types in 2001. From these two studies, it was established that healthcare facilities did not have the proper means of managing healthcare wastes [13]. Following these studies, about 13 pilot small-scale incinerators were built in several areas in Tanzania. Following good performance of these pilot incinerators, it was recommended to expand the programme by building more incinerators in all referral hospitals and regional and district hospitals accompanied by training of the healthcare staff [23]. However, for incinerators to work efficiently and effectively, the management and handling of healthcare wastes must be improved, which includes separation of wastes. The objective of this work was to examine healthcare solid waste management systems in Shinyanga municipality as a case study.

## 2. Methodology

### 2.1. Description of the Study Area

**Figure 1:**
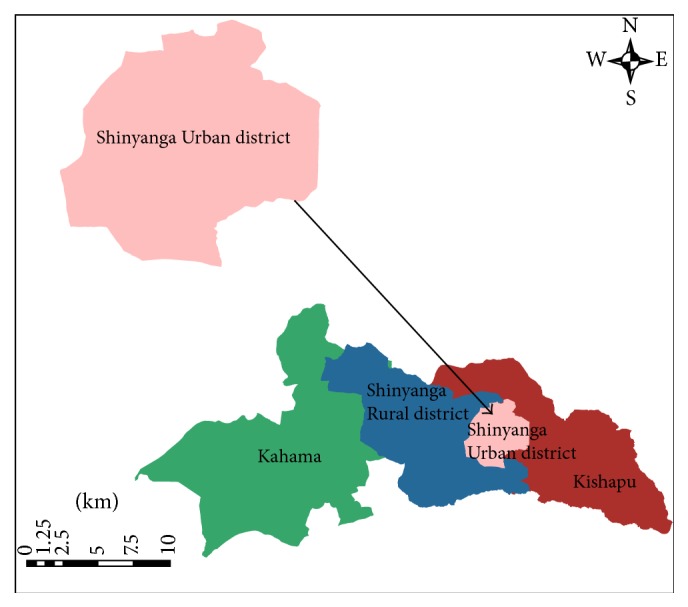
Map of Shinyanga Region showing location of the study area.

Shinyanga Region, which is located about 30 to 60 km south of Lake Victoria between Latitudes 31°14′ and 35°11′E and Longitudes 2°15′ and 4°30′S, is administratively divided into 4 districts of Kahama, Kishapu, Shinyanga Rural District, and Shinyanga Urban District. The region occupies an area of 18,555 km^2^ with a population of approximately 1,277,181 people. Shinyanga municipality with 13 wards is located in the Shinyanga Urban District, which is the smallest district in the region (Figure 1). According to the 2002 Tanzania National Census, the population of the municipality is 135,166 in 17,702 households. The municipality has 11 healthcare facilities, which are Shinyanga Regional Referral Hospital (RRH), Kambarage Urban Health Centre (UHC), Kolandoto District Designated Hospital (DDH), and others which are dispensaries, namely, Kizumbi, Lubaga, Chamaguha, Old Shinyanga, Ibadakuli, Galamba, Mwawaza and Mwamalili.

The study was conducted in Shinyanga Municipal Council covering three major hospitals, which are Shinyanga RRH, Kolandoto DDH, and Kambarage UHC. These healthcare facilities were chosen because they are the leading health institutions in the municipality. The hospitals are comprised of departments, like wards, the casualty, the minor operating theatre, the major operating theatre, maternity section, laboratory, and X-ray section among others.

### 2.2. Study Design and Data Collection

The primary data were collected using questionnaire, interviews, and observation checklists and secondary data were obtained from the hospital documents. In accordance with data from the three healthcare facilities, Shinyanga RRH has a staff strength of 229, Kolandoto DDH has 141 employees, and Kambarage UHC has 30 employees. The sample size for this study was determined in accordance with (1), which was developed by Krejcie and Morgan [24]:

(1) $$\begin{array}{c} n=\frac{Z^{2}\times p\times q\times N}{e^{2}\left(N-1\right)+Z^{2}\times p\times \left(1-p\right)}, \end{array}$$

where *N* is total population size = 49, *n* is sample size, *Z* is confidence level = 95%, and *p* is proportion of the population = 0.5. The sample size for a margin of error *e* of 5% is for Shinyanga RRH, Kolandoto DDH, and Kambarage UHC 93, 58, and 12 employees, respectively. A total of 207 employees participated in the study, which included 127 from Shinyanga RRH, 60 from Kolandoto DDH, and 20 from Kambarage UHC (Table 1). This sample population size was 27% more than the sample population size of 163 required by the study owing to the willingness of employees to participate in the study. The sample size was selected systematically from healthcare workers who are willing to participate in the research from each hospital. This included individuals who participated in the research and who were obtained based on their administrative responsibilities.

Three tools were employed to collect data for the study, namely, observation checklists, interviews, and questionnaires. Observation checklist was used to gain intimate familiarity and some insights concerning the healthcare wastes management practices, health workers' perceptions and their involvement, and perceptions and knowledge as well as adherence to healthcare waste management policies and regulations. They were used to collect and record information from the health workers on the amount of healthcare wastes generated by hospitals, to check adherence to waste segregation, collection mode and frequency and technical matters such as storage facilities available at the hospitals, and transportation and final disposal of the wastes. Checklists were also used to record information regarding problems encountered by the hospitals in the management of healthcare wastes as provided by the informants and other health workers.

### 2.3. Generation of Healthcare Wastes

The major category of healthcare wastes was collected and measured include general (food stuffs, papers, etc.) and clinical wastes (pathological, sharps, infectious, pharmaceutical, and radioactive wastes). In absence of effective wastes segregation facilities within the healthcare facilities, the daily quantities of wastes were measured for 5 weeks at the point of collection before wastes were disposed of. Clinical wastes were largely found in maternity wards, the casualty department, the operating theatres, laboratory, and X-ray section among others. Maternity wards were the major contributor to pathological, infectious, and sharp wastes owing to the nature of the wards.

The number of patients in the wards was determined on a daily basis before collecting the wastes. Wastes from maternity wards were collected from delivery rooms and all mothers in maternity wards were asked to put the wastes in the provided buckets. Shinyanga RHH and Kolandoto DDH have 86 and 24 maternity beds, respectively, although only about 18 beds are occupied on average at Kolandoto DDH because cost of healthcare services are prohibitive to some patients. The amount of healthcare wastes generated from wards was determined by segregating and measuring the weight of wastes collected in the plastic buckets from the wards every day at 06:00 a.m. for the period of five consecutive days. The weight generated was measured using a digital weighing scale and was recorded on the checklist on a daily basis for five days of the exercise. The average amount of healthcare solid wastes generated per person per day was determined for each of the two hospitals involved. This was done by taking the amount (kg/day) generated by the ward dividing it by the number of patients in the wards. Nurses on duty were trained to monitor and supervise the patients to ensure that wastes are put in the provided bucket.

Wastes generated from other sources including administration units and landscaping were segregated, measured, and recorded separately. This was done every morning at 06:00 a.m. before emptying the bins. The sorting was done by collecting different categories of healthcare wastes into containers with different colors. The colored containers were blue, red, and yellow for noninfectious, infectious, and sharps wastes, respectively. The average amount of wastes generated by each hospital was determined. The weight of empty containers and plastic buckets were predetermined before using them for wastes storage.

### 2.4. Interviews and Questionnaires

Interviews were conducted with key informants in the institutions with the help of an interview guide, which was used to get in-depth information and deeper insights on the hospital profiles such as year of establishments, number of beds, number of wards, staff strength, average number of in-patients and outpatients, and annual budget. They were also used to gather information on the type and quantity of healthcare solid wastes generated, the existence of healthcare wastes policies in the hospitals, training (if any) provided for healthcare facilities staff on healthcare solid wastes management, practices on healthcare solid wastes management (generation, segregation, collection, storage, transportation, treatment, and disposal) and specific healthcare wastes management budget, and other financial arrangements at the sampled hospitals as well as perceptions, attitudes, opinions, and understanding of regulations and guidelines for managing healthcare wastes.

A total of 9 interviewees participated in the study, including 4, 3, and 2 from the Shinyanga RRH, Kolandoto DDH, and Kambarage UHC, respectively. Key informants were sampled from the hospitals administration staff who have in-depth knowledge and responsibilities of healthcare wastes management practices and laws, policies, and regulations. The key informants included environmental health officers, healthcare officers, nurse in charge, heads of departments, cleaners at the hospitals, and the crew that ferried healthcare waste to the incinerator. They gave their knowledge and experiences on the healthcare wastes management practices and enforcement of laws and regulations as well as implementation of the policies. Multiple informants were used to increase the reliability and validity of information. An interview guide was prepared for each one of them. This confirmed and strengthened information collected during site visits. Interviews were conducted with the personnel responsible for environmental healthcare in each hospital and with the personnel involved in the collection, handling, and disposal of hospital wastes.

The questionnaires were administered to the health workers to gather primary data on the current healthcare waste management practices including mode and frequency of waste collection, availability of resources for waste management, and challenges of managing the final disposal of waste in the sampled hospitals. Unlike interview which was conducted for administrators and heads of departments and sections, questionnaires were developed for healthcare staff such as doctors, nurses, laboratory technicians, environmental health officers, and healthcare solid waste personnel. They were self-administered to respondents in the hospitals and collected as agreed upon. The sections that were involved in the study are pharmacy, surgery, injection and wounds, maternity wards, obstetrics, and gynecology.

### 2.5. Data Analysis and Presentation

Data regarding the demographic information of respondents, the profile of the sampled hospitals, and the current situation of healthcare wastes management practices were extracted from questionnaire, observation checklist, and interviews. The Microsoft Office Excel and Statistical Package for Social Sciences (SPSS version 16.0) programmes were used to analyze the data. Presentation of the outputs was done using tables.

## 3. Results and Discussion

### 3.1. Profile of the Hospitals

Shinyanga Regional Referral Hospital, which is the major health facility in the region, was established in 1947 as a health centre. It was officially promoted to regional hospital in 1974. The vast majority of patients using this hospital are local poor residents. It was designed to accommodate 333 in-patients, but during the study it had 356 in-patients in the 12 wards. An additional 184 outpatients receive medical services daily at this hospital. Since 2010, this has been serving as regional referral hospital with staff strength of 229 and an annual budget of about 1.2 billion shillings.

Kolandoto DDH is a nongovernmental institution, which was founded in 1913 by missionaries from Africa Inland Church. The hospital, which has 182 beds in 7 wards and maternity section with 2 delivery rooms and 24 beds, is located about 16 kilometers northeast of Shinyanga municipality, along Shinyanga-Mwanza road. It was promoted to district designated hospital in 2010 to serve as a district hospital, and it is currently managed jointly by the municipality and Africa Inland Church of Tanzania. Kolandoto DDH has 141 staff, with annual budget of about 800 million shillings and about half of the budget is received from the government.

Kambarage Urban Health Centre is an outpatient health facility located at Kambarage ward within the municipality. It was built in 2010 to serve as a subdistrict hospital after upgrading of the Shinyanga Regional Hospital to regional referral hospital and the Kolandoto hospital to District Designated Hospital. The hospital has 30 employees that serve about 104 outpatients daily.

### 3.2. Demographic Information of the Study Participants

The study participants were comprised of nurses, environmental health officers, medical attendants, and doctors of different positions in the sampled hospitals, who were willing to participate in the study (Table 2). Of the 127 respondents at Shinyanga RRH, 75 (59%) were medical attendants, 36 (28%) were nurses, 12 (10%) were environmental health officers, and 4 (3%) were doctors, which is similar to proportions of the health professionals in the hospital. At Kolandoto DDH the respondents involved in the study were 38 (63%) medical attendants, 13 (22%) nurses, 7 (12%) environmental health officers, and 2 (3%) doctors. For Kambarage UHC respondents were 8 (40%) medical attendants, 9 (45%) nurses, 1 (5%) environmental health officer (EHO), and 2 (10%) doctors.

It was observed that the age of the respondents was fairly distributed between age groups of 31 to 45 years (44.4%) and 46–60 years (44.9%). Only 2 (1.0%) of the participants were above 60 years and 20 (9.7%) were below 31 years. In terms of gender, 56% of the respondents were men and 44% were women. Male respondents were more dominant at Shinyanga RRH with 59.8% participants, but female respondents were more dominant at Kambarage UHC with 60% participants. About 67.1% of the respondents have completed tertiary education, while only 8.2% are primary school leavers. The proportion of primary school leavers varied from as low as 7.1% at Shinyanga RRH to as high as 15% for Kambarage UHC. The reverse was observed for tertiary education where 60% of the respondents have completed tertiary education at Kambarage UHC rising to 69.3% for Shinyanga RRH, which corresponds to the level of service provided by these health facilities. About 91.8% of the respondents have completed secondary education and are therefore capable of reading and filling in the questionnaires without problems.

### 3.3. Knowledge of Employees of the Classification Healthcare Wastes

The healthcare workers at the sampled hospitals seem to be aware of the type and the hazardous nature of healthcare wastes. This is because of their familiarity with syringes and needles and the accidents that might happen as a result of sharps injury. However, some confusion exists among workers especially with chemicals, unused medicines, and pressurized containers. It was observed that 47% of the workers considered pressurized containers as healthcare wastes (Table 3). On the other hand, 77% of the respondents considered chemicals as healthcare wastes. In the same vein 89% and 97% of the employees considered dressing cotton and pharmaceutical wastes as healthcare wastes. These wastes are not healthcare wastes and should not be confused with other healthcare wastes.

On the other hand, the paper, carton, and boxes were classified as healthcare wastes by 24% of the respondents. It was further observed that 33% of employees involved in the study considered kitchen wastes as healthcare wastes. Some of health sector employees responded that chemicals (23%), pathological materials (10%), and unused medicines (15%), respectively, are not healthcare wastes. It is therefore clear that even when separation of wastes is practiced, wastes are likely to be mixed because employees cannot distinguish healthcare wastes from general wastes. Tiong et al. [25] in their survey of 19 private healthcare clinics in Malaysia observed that 57.9% of the private clinics were practicing improper management of healthcare wastes because of lack of awareness.

It was further revealed that 44 of the 58 nurses (76%) classified the wastes correctly. However, only 2 of the 8 doctors (25%), 40 of 121 medical attendants (33%), and 9 of 20 environmental health officers (45%) completed correct classification. It therefore appears that nurses have more knowledge of type of wastes than other healthcare workers probably because they are regularly involved with waste management practices.

### 3.4. Knowledge of Policies, Laws, and Regulations Regulating Healthcare Waste Management

Generally, there was very low level of awareness of existence of documents regulating healthcare wastes and by extension the environment, among the respondents. It was observed that only 16.9% of the respondents knew about the existence of the WHO manual on safe management of wastes from healthcare activities [26]. Only 17.9% and 13.5% of the respondents had the knowledge of the existence of the Environmental Management Act [27] and the Public Health Act [28], respectively. It was surprising to learn that 51.7% of health workers including those in the top administrative positions were not aware of the existence of any one of the three documents. Among the interviewed administrative staff, the national healthcare policy, Environmental Management Act [27], and Public Health Act [28] were particularly well known by only 2 of the 9 respondents (22.2%), but no one was sure where these documents are kept. In general, higher age groups (experienced) people were relatively keener on improving the waste management practices whereas most of the employees in the younger age group were relatively unconcerned with waste management. A similar behavior was reported by Denniss [29] who observed that young generations were less aware of the environmental issues and are less concerned with waste management.

Elsewhere, Kaiser et al. [30] in a study in the United States reported a gap on awareness of environmental issues in general by hospital workers, which negatively affects and influences the choice of materials used in hospitals. A case study conducted by Patil and Pokhrel [31] in a hospital in India found that the pockets of noncompliance with statutory requirements were due to a lack of enforcement. Policies, acts, regulations, and codes of practice contain information that justifies their formulation and they emphasize the importance of the issue they regulate. It is therefore absolutely important that those who implement them are familiar with their contents and requirements.

### 3.5. Provision of Training and Education on Management of Healthcare Wastes

Results from the questionnaires showed that training was not provided to the waste management staff, doctors, and other personnel on management of healthcare wastes and their potential hazards. It is worth reporting that 88.2% of Shinyanga RRH employees received no training on management of healthcare wastes. It was also observed that 90% and 95% of employees of Kolandoto DDH and Kambarage UHC, respectively, received no formal training. Even employees from municipal council who are responsible for collection and disposal of the same wastes have not received formal training on management of healthcare wastes and are consequently unaware of the environmental health impacts of these wastes. On the other hand, those who indicated that training was provided could not tell the contents of training and were unable to remember when and where they were trained, which is an indication that no training was provided. However, 79% of the health workers were very positive on training needs for management of healthcare wastes, but the remaining respondents either were not concerned with training (22%) or considered training as unimportant (8%).

In accordance with administrators, the major reasons for lack of training were budget constraints (67%), lack of skilled trainers (23%), and lack of willingness to provide training (10%). On the other hand, other health workers pointed out that financial constraints (80%) were the main reason for not providing training on healthcare waste management in their respective hospitals although they also identified other reasons such as lack of skilled trainers (17%) and lack of willingness to provide the training (3%). It is evident that lack of training in the hospitals with respect to management of healthcare wastes poses serious risks to the health of personnel. Continuous training of hospital staff is an important way of ensuring that knowledge is enhanced among environmental health practitioners, nurses, and doctors in particular, about the management of healthcare waste. Healthcare wastes are not only a reservoir of pathogenic organisms but also an important source of hospital acquired infections (HAIs) and hence a key component of the infection control programmes [4].

Botelho [32] reported that, to effectively manage healthcare wastes, provision of education and training is the strongest factor influencing degree of compliance to healthcare management procedures and regulations. By building a strong knowledge base among healthcare workers, they will engage in practices that protect them and their patients as well as the communities and the environment. The knowledge of the constituents of healthcare waste in the sampled hospitals is generally considered as one of the main barriers towards proper healthcare waste management [33]. Patwary et al. [18] in their study in Dhaka city, Bangladesh, observed that healthcare waste handlers were frequently found to be untrained and lacked even a basic understanding of the hazards involved. This is similar to observations made in this work and elsewhere in Tanzania. In accordance with Manyele and Anicetus [34], a survey of hospitals in eight regions in Tanzania suggested that there was low knowledge of healthcare wastes among staff, as well as use of untrained casual laborers to handle healthcare wastes. Even among professionals, the need for training cannot be ignored. In accordance with Matiko [3] about 96.7% of the contacted medicines store supervisors wanted professional training on pharmaceutical disposal so as to impart them with more exposure to enhance their knowledge of pharmaceutical management skills of the medicines. He further observed that about 63.3% of the store supervisors mentioned handling unwanted medicines for disposal as the key area of training required.

### 3.6. Provision of Personal Protective Equipment to Waste Workers

An overwhelming majority of the health employees who participated in the study (81%) are not using personal protective equipment (PPE), which include gum boots, gloves, caps, and overall coats when handling healthcare wastes. The remaining 19% of the respondents reported that they are using at least gloves, and they are using gloves they purchase using their own money because employers do not provide them with this important protective gear. Provision of protective gears to all healthcare workers is important for reducing potential health risks to them. Gloves are just one of the forms of protective gear that is used to prevent direct contact with healthcare waste in order to reduce the risk of infection [35].

In accordance with Ministry of Health and Social Welfare (undated) the use of PPE is now more important than ever before because of the emergence of HIV/AIDS and Hepatitis B and Hepatitis C infections and the resurgence of tuberculosis in many countries. However, inadequate budgets have left healthcare facilities with inadequate and inconsistent supply of PPE. As a result the availability of plastic aprons, boots, and heavy-duty gloves in the primary healthcare facilities surveyed in Ilala municipality was only 10%, 25%, and 40%, respectively [36], which is suggesting the observation made in Shinyanga is not uncommon practice in Tanzania.

### 3.7. Healthcare Wastes Generation Rate

The average daily healthcare waste generated was determined from the wards and other sources. Beds that were not occupied during the study were not involved in the calculation of healthcare wastes generation rates. At Shinyanga RRH it was observed that the generation rate is 569 kg/day (Table 4). The generation by category indicates that general wastes amount to 508 kg/day, followed by pathological wastes (43 kg/day), infectious wastes (12 kg/day), and sharp wastes (6 kg/day). At Kolandoto DDH it was observed that general wastes were 95 kg/day followed by pathological wastes (18 kg/day), infectious wastes (4 kg/day), and sharp wastes (2 kg/day). These figures are translating to hazardous waste generation rate of 171 g/in-patient/day at Shinyanga RRH and 264 g/in-patient/day at Kolandoto DDH. At Kambarage UHC the results show that general wastes were 82 kg/day, pathological wastes were 0.5 kg/day, infectious wastes were 2 kg/day, and sharp wastes were 0.5 kg/day. Low quantity of infectious and pathological wastes at Kambarage UHC was because of its nature of activities. Tiong et al. [25] reported that 57.9% of the medical clinics generated less than 1 kg of human tissues/blood/fluids waste per day and only 1 medical clinic generated 1–5 kg of human tissues/blood/fluids waste per day. Therefore these results were not unexpected.

It was revealed that average daily solid waste generation per patient at Shinyanga RRH and Kolandoto DDH was 1.6 kg/patient/day and 1.3 kg/patient/day, respectively (Table 4). These values are within the range of 0.3–1.8 kg/day reported by Mato and Kassenga [37]. Elsewhere, Hamoda et al. [38] reported that, in Middle East, Latin America, and India solid wastes generation rate ranges between 1.0 and 3.0 kg/day. In Turkey, Soysal et al. [2] carried out a cross-sectional study with 825 health instructions of Izmir metropolitan municipality and observed solid waste generation ranging from 0.32 to 2.79 (mean 1.7) kg/patient/day.

Since Kambarage UHC is an OPD health facility, the amount of solid wastes generated from the hospital wards could not be determined because it has no wards. It is evident that Shinyanga RRH produces more waste per bed than Kolandoto DDH, which is in line with the fact that urban hospitals generate more wastes per bed because of high standard of living. Although both hospitals are in the same municipality, Kolandoto DDH is in the rural setting unlike Shinyanga RRH which is in the town centre. The fraction of sharps wastes is relatively small but poses most of the risk to health workers and community especially on needle stick injuries necessitating more attention [39]. Currently, such wastes are collected in safety boxes supplied by Ministry of Health and Social Welfare.

Table 4 shows that the fraction of hazardous wastes (i.e., pathological, infectious, and sharps wastes) varies from 4% of the total wastes at Kambarage UHC to 11% at Shinyanga RRH and 20% at Kolandoto DDH. Small propositions of hazardous wastes were generated at Kambarage UHC compared to large hospitals because of the nature of healthcare facilities. Hospitals with in-patients perform large operations and have maternity wards, which generate large quantities of infectious wastes. Elsewhere, Rahman [40] have reported comparable proportions of hazardous wastes ranging from as low as 5% in the Netherlands to as high as 16% in Bangladesh. The proportion of hazardous wastes in Sweden and Germany are 9% and 14%, respectively. In Malaysia, Tiong et al. [25] reported that proportion of hazardous wastes is 19% of the total wastes and WHO [7] estimated that only 20% of the total healthcare wastes are infectious. The variation of the proportion of hazardous wastes may be due to a number of reasons including differing living habits and standards, availability of different treatment facilities, geographical location, and perhaps the ways in which healthcare wastes are segregated and categorized in the different countries.

### 3.8. Waste Segregation and Color Coding of Collection Containers

Careful segregation of wastes into different categories helps to minimize the quantities of hazardous waste. A proper segregation is expected to identify wastes according to their source and type of disposal or disinfection. The hospitals are responsible for provision of receptacles specifically suited for each category of wastes, which are identified by color-codes and appropriate labels. In accordance with MoH [41, 42], the segregation must take place at the source of generation such as at the ward bedside, operation theatre, diagnostic laboratory, or any other room or ward in the hospital where waste is generated. In accordance with WHO [26] recommendations, hospitals have to provide plastic bags and strong plastic containers for infectious wastes such as empty containers of antiseptics used in the hospital. Bags and containers for infectious wastes should be marked with biohazard symbol [4].

However, when health workers were asked to indicate if storage bins for the different types of healthcare wastes were available in their hospitals, the vast majority (96%, 90% and 85%) at Shinyanga RRH, Kolandoto DDH, and Kambarage UHC, respectively, reported that separate bins were not available. When asked if they knew how to distinguish the various types of bins for the storage of different types of healthcare wastes, 97% at Shinyanga RRH, 83% at Kolandoto DDH, and 91% at Kambarage UHC reported that they did not know. The results revealed that the employees had no knowledge of the availability of separate bins for the different types of healthcare risk wastes in the wards and the hospitals in general and had not seen how the bins look like. This is because the hospital management has not labeled infectious waste bins with biohazard symbols.

It was observe that all three health facilities do not separate healthcare waste from general waste stream at the waste production points. In the wards, doctors and nurses who use sharps are required to drop them into different containers, but this is not diligently followed. In one occasion, used sharps were left on hospital bed at Shinyanga RRH, which could be dangerous to patients. As the wastes are generated in all the departments of the healthcare facility, it is handled by the medical staff; cleaners, gardeners, laborers; refuse attendants and even watchmen are sometimes responsible for burning of the wastes. Previous studies in Tanzania have made similar observations (World Bank and MoH, 2003) [34]. In light of the above findings, it was revealed that segregation of healthcare wastes into infectious and noninfectious wastes is not conducted in accordance with regulations and standards.

### 3.9. Handling, Treatment and Disposal of Healthcare Wastes

Healthcare wastes generated by the three hospitals are on daily basis collected and transported from the offices, wards, and theatres to temporary storage areas or disposal sites by hospital staffs by means of wheeled trolleys or containers. The majority (81%) of staff employed for handling these wastes in the hospitals did not have appropriate personal protective equipment (PPE), including overall gowns, protective boots, and gloves. It is important to note that the lack of suitable and sufficient protective equipment, the lack of knowledge regarding the correct usage of equipment, and the lack of pertinent understanding of the personnel regarding the benefits of using protective equipment expose personnel to serious dangers [43].

It is noteworthy to report that all three hospitals do not maintain records or register for healthcare waste disposal and have insecure, poorly managed temporary storage areas that are not fenced. Kolandoto DDH and Kambarage UHC use these areas for burning healthcare solid wastes, but Shinyanga RRH transport healthcare wastes to municipal dumpsite. The infectious and noninfectious wastes are kept in the hospital's own temporary storage area before being collected by municipal truck for final disposal at Nhelegani dumpsite. The wastes are loaded directly into the municipal trucks without putting them first into closed separate containers, which may pose serious health risks to workers managing these wastes as well as general public as wastes may fall off on the roads during transportation or infect waste pickers at the dumpsite. In accordance with Johannessen et al. [9] it is recommended to transport healthcare waste on public roads in closed containers in dedicated vehicles and wastes must be handled by trained staff.

Waste treatment leads to a decrease in volume, weight, and risk of infectivity and organic compounds of the waste [4]. Incineration is the main method for treatment of healthcare waste especially infectious and sharp wastes at Shinyanga RRH and Kambarage UHC, but healthcare wastes are burnt at Kolandoto DDH because their incinerator is out of operation. In accordance with Ministry of Health and Social Welfare [44], the main disposal methods of healthcare wastes in the hospitals were through burning (50%) and burying (30%) of wastes. These methods were also widely used in all health facilities in Shinyanga, particularly at Kambarage UHC and Kolandoto DDH. It is not uncommon to find healthcare facilities which do not possess incinerators, because in accordance with Manyele and Anicetus [34] Tanzania has low incineration capacity for treatment of healthcare wastes. In fact National Bureau of Statistics and Macro International Inc. [45] have reported that the proportion of hospitals, health centres, and dispensaries with adequate disposal system for infectious wastes were only 48%, 34%, and 28%, respectively.

**Figure 2:**
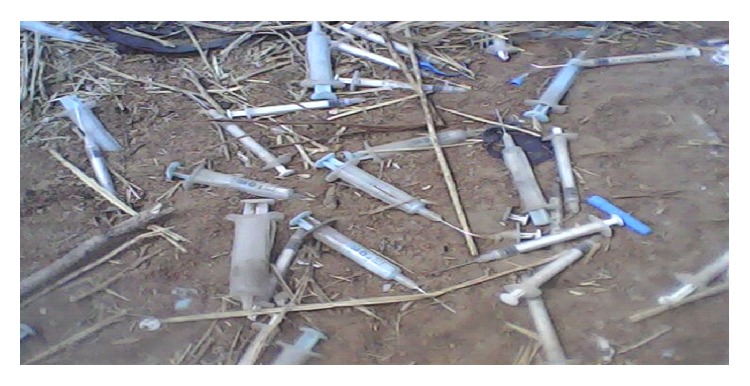
Syringes and needles in a municipal solid waste dumpsite at Nhelegani.

About 11% of wastes at Shinyanga RRH were incinerated without segregation in locally built incinerator lined with bricks, but some healthcare wastes still find their way to Nhelegani municipal solid waste dumpsite along with the remaining 89% of the general wastes. This poses risks to people and environment as it can be source of infection and pollution to underground water and also destruction of the flora and fauna [41, 42]. The quality and availability of disposal facilities for healthcare wastes are generally poor and inadequate systems for disposal of infectious waste, including sharps, were clearly evident. The risk is particularly high when healthcare wastes are disposed of together with general wastes, which may be a cause of transmission of diseases amongst waste pickers, recycling waste operators, cleaners, and waste collectors [46]. Such risks may be evident from haphazardly dumped syringes and needles in a municipal solid waste at Nhelegani dumpsite (Figure 2).

The application of incineration for treatment of healthcare waste is considered favorable option by 71% of the health employees who participated in the study. Other preferred options are chemical (16%), land disposal (8%), and autoclaving (5%) options, although some respondents could not clearly describe how some of these technologies work. In accordance with Patwary et al. [18] incineration and autoclaving are preferred technologies for treatment of healthcare wastes in developed countries. While many countries are maintaining stringent healthcare wastes management systems to minimize health risks to healthcare workers and the general public [47, 48], healthcare wastes are not receiving adequate attention in developing countries, with this particular case inclusive.

### 3.10. Institutional Set-Up Deficiencies

It is possible that inadequacies in laws and regulations have left room for such malpractices. For example, the conditions for final disposal of healthcare solid wastes are set in Section 137, subsections 1 and 2, of the Environmental Management Act of 2004 [27]. However, regulations to enforce this law have not yet been established. In addition, the existing Public Health Act has no specific clauses on healthcare waste management, which allows room for unsatisfactory, inconsistent, and unregulated healthcare waste management practices. The majority (83%) of the health workers proposed the establishment of subcommittees for monitoring and supervision of the final disposal of the healthcare wastes. Such subcommittees do not exist at the moment, but municipal councils have health department, supposed to handle such matters. However, municipal councils have health officers who among other duties are supposed to follow up management of healthcare wastes including monitoring and promotion of awareness.

Health facilities have also failed to allocate financial resource for human resource development for capacity building and adequate personal protective equipment for waste handlers in the sampled hospitals. A report by Ministry of Health and Social Welfare [44] admitted that health facilities are faced with the challenge of a constant supply of PPE because of inadequate budget, although other numerous factors contribute to their inconsistent use even when available. Interventions to these deficiencies might involve reviewing of the policies, legislation, organizational structures within healthcare facilities, municipal councils, or Ministry of Health and Social Welfare in order to reduce risks to the waste handlers and general public.

It is understood that there is a national healthcare waste management plan, which was established by Ministry of Health and Social Services in 2006 to enhance healthcare waste management options and to provide guidance for healthcare facilities in developing their policy and procedures [41, 42]. The National Healthcare Waste Management Programme [49] under the Directorate of Preventive Health Services prepared a six-year national action plan that was supposed to be implemented from 2009 to 2015. This action plan was intended to address the majority of the issues discussed in this work. Unfortunately, this action plan has not been implemented at user level (healthcare facilities) countrywide, which could prove to be very resourceful if implemented at national level down to the healthcare facility level.

## 4. Conclusions

From the results of this research, the following conclusions are made:

1. The total quantities of solid waste generated in the three hospitals are 569, 119, and 85 kg/day for Shinyanga RRH, Kolandoto DDH, and Kambarage UHC, respectively. The rate of generation of waste was about 1.6 kg/in-patient/day for Shinyanga RRH and 1.3 kg/in-patient/day for Kolandoto DDH. The fraction of hazardous wastes varies from 4% of the total wastes at Kambarage UHC to 11% at Shinyanga RRH and 20% at Kolandoto DDH. The nature of healthcare facility was observed to influence the proportion of the hazardous wastes in the total wastes.
2. Healthcare wastes are not segregated from the general wastes. As a result they are mixed with general wastes, which poses risk of infection to healthcare worker and the general public. In general, healthcare wastes are managed improperly and inconsistently and do not comply with Environmental Management Act [27], Public Health Act [28], and WHO recommended guidelines.
3. To effectively manage healthcare wastes, the provision education and training to healthcare workers were considered an important requirement. By building a strong knowledge base among healthcare workers, they will engage in practices that protect them and their patients as well as the general public and the environment.

## Figures and Tables

**Table 1:** Employees participating in providing information during the study.

Professional	Hospital			
	Shinyanga RRH	Kolandoto DDH	Kambarage UHC	Total
Nurses	36	13	9	58
Doctors	4	2	2	8
Medical attendants	75	38	8	121
Environmental health officers	12	7	1	20
Total	**127**	**60**	**20**	**207**

**Table 2:** Demographic information of employees in the sampled hospitals.

Item	Characteristics	SRRH		KDDH		KUHC		Total	
		Number	%	Number	%	Number	%	Number	%
Age	16–30	10	7.9	7	11.7	3	15.0	20	9.7
	31–45	58	45.7	28	46.7	6	30.0	92	44.4
	46–60	57	44.9	25	41.6	11	55.0	93	44.9
	61 and above	2	1.5	0	0	0	0	2	1.0
	Total	127	100	60	100	20	100	207	100

Gender	Male	76	59.8	32	53.3	8	40.0	116	56.0
	Female	51	40.2	28	46.7	12	60.0	91	44.0
	Total	127	100	60	100	20	100	207	100

Level of education	Primary	9	7.1	5	8.3	3	15.0	17	8.2
	Secondary	30	23.6	16	26.7	5	25.0	51	24.6
	Tertiary	88	69.3	39	65.0	12	60.0	139	67.1
	Total	127	100	60	100	20	100	207	100

**Table 3:** Knowledge of employees of classification of wastes.

Type	Respondents who considered wastes as healthcare waste (%)
Paper, cartons, and boxes	24
Dressing cotton and plasters	89
Chemicals	77
Pathological materials	90
Pharmaceuticals	97
Unused medicines	85
Kitchen wastes	33
Pressurized containers	47

**Table 4:** Summary of healthcare waste generation rates for SRRH, KDDH, and KUHC.

Generation rate	SRRH	KDDH	KUHC
Total wastes (kg/day)	569	119	85
Number of beds	333	102	—
Daily in-patients (patients occupying a bed)	356	91	—
Waste generation rate (kg/patient/day)	1.6	1.3	—
